# ST segment depression: the possible role of global repolarization dynamics

**DOI:** 10.1186/1475-925X-6-6

**Published:** 2007-02-09

**Authors:** Bruce Hopenfeld

**Affiliations:** 1 Angel Medical Systems, 1 Sheila Drive, Tinton Falls, New Jersey 07724, USA

## Abstract

**Background:**

At least some clinical data suggests that, regardless of which major coronary artery is narrowed, the early ST segment body surface pattern is characterized by a minimum near precordial lead V5 and a broad area of left precordial negative potentials. Some clinical data also suggests that late ST segment potentials can localize an ischemic heart region.

**Objective:**

A computer model of a heart/torso system was implemented to study the relationship between transmembrane potentials throughout the heart and clinically observed body surface potential patterns during the early and late ST segments in ischemic patients.

**Methods:**

Transmembrane potentials were selected to produce body surface potentials that matched the clinical data.

**Results:**

The early ST segment pattern was matched by assigning: (i) an epicardial transmembrane potential gradient that is consistent with the normal activation/repolarization sequence, according to which the left lateral epicardium activates relatively late; (ii) an endocardial transmembrane potential distribution with the lowest transmembrane potentials in the ischemic region; and (iii) overall lower transmembrane potentials to the endocardium compared to the epicardium. Late ST segment potentials, which localized the area of the ischemic region, were generated by reducing the epicardial transmembrane potential gradient and increasing the endocardial transmembrane potential gradient.

**Conclusion:**

The non-localizing nature of early ST segment depression could be due to global epicardial and endocardial transmembrane potential gradients related to the activation/repolarization sequence, whereas the possibly localizing nature of late ST segment depression could be due to the relative removal of the epicardial gradient, and an increase of the transmembrane potential gradient across the endocardium.

## Background

ST segment changes have been associated with ischemia for over 3 decades and are clinically accepted as indicators of ischemia. Acute, focused ST elevations have been shown in both human and animal models to result from transmural ischemia. Diffuse ST depressions, on the other hand, have been associated with subendocardial ischemia, although the mechanisms underlying the diffuse pattern have not been firmly established. This paper continues to expand on this important issue in the hopes of providing greater insight into the relationship between what is occurring in the heart and what is detected on the body surface. This paper will address biophysical processes that may underlie ST segment changes caused by subendocardial ischemia. These processes were simulated with a computer model of a heart/torso system, and the results of these simulations will be presented.

It is generally accepted that ST segment depression, as conventionally measured approximately 60 ms after the J point, does not localize the site of subendocardial ischemia[1]. According to the solid angle theory, which presupposes electrically isotropic tissue (or tissue characterized by equal anisotropy ratios), ST depression should occur over the ischemic region[1]. Since this does not appear to occur in reality, alternatives to the solid angle theory have been advanced. In particular, some computer modeling studies have attributed the inability to localize subendocardial ischemia to anisotropic characteristics of heart tissue[1-3].

**Figure 1:**
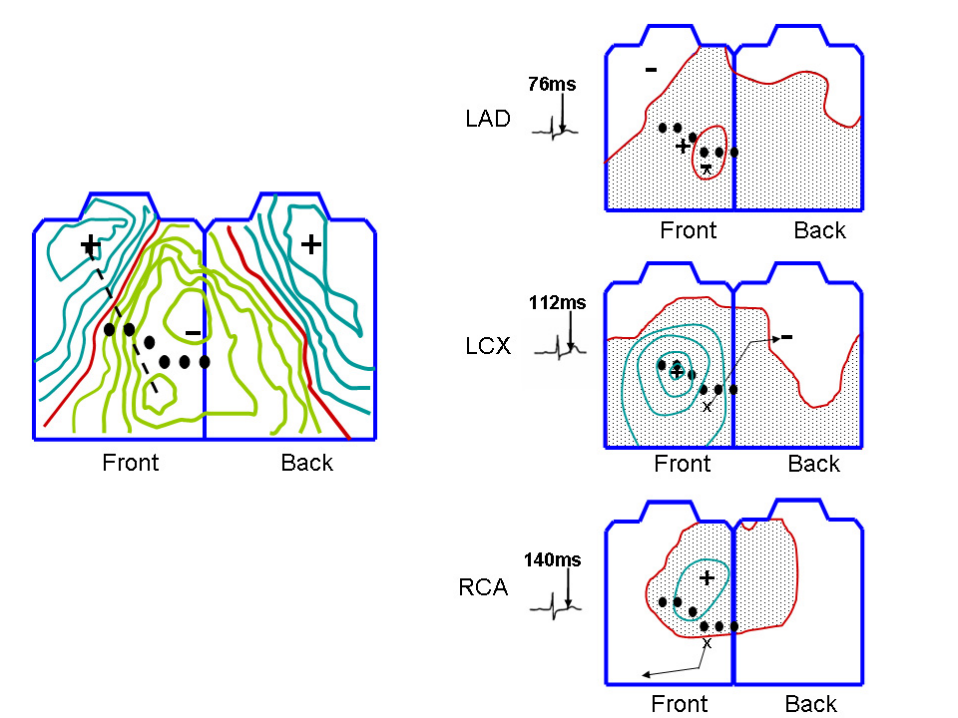
Clinical body surface maps. The left hand panel shows a difference map between patients with LAD occlusions and healthy subjects adapted from Hanninen et al.[4] Positive potentials are shown in aqua, negative potentials in green and the 0 line in red. Preocordial lead positions are indicated by filled black circles. The right hand panel shows body surface maps adapted from Kubota et al.[6] in the case of LAD, LCX and RCA occlusions. The color scheme is identical to the left hand panel. The isopotential contour interval is 0.1 mV. The time the map was recorded, measured with respect to the J point, is indicated by the electrogram to the left of each map. The "x" in each map indicates the site of the minimum during the early ST segment, and the arrow indicates the movement of the minimum throughout the ST segment. See text for additional details.

Although solving some inconsistencies between what is expected and what is clinically seen, these previous studies have not attempted to reproduce torso potentials that match clinically observed early ST segment potentials associated with single vessel disease of all three major coronary arteries. In particular, these models do not predict a potential minimum in the lower left precordium and a potential maximum over the sternum in the case of occlusions of the left anterior descending artery (LAD), left circumflex artery (LCX) or right coronary artery (RCA)[4]. Moreover, these computer models do not predict the observed widespread relatively negative potentials in the lower left of the torso and positive potentials over the upper right torso in the case of occlusions of the LAD, LCX or RCA[4]. The left hand panel in Figure 1, a clinical body surface map[4] of the difference between LAD diseased patients and normal subjects, illustrates some aspects of the above mentioned early ST segment patterns.

The present study investigates a new hypothesis, based on a three dimensional computer heart/torso model and biophysical arguments, that clinically observed early ST segment potential distributions caused by ischemia *could *have resulted from a combination of global transmural and global epicardial transmembrane potential gradients. These global transmembrane potential gradients could have been a consequence of the activation/repolarization sequence, which can cause earlier activated tissue to be more repolarized than later activated tissue during the early ST segment. Indeed, Aslanidi et al. [5] showed that in a one dimensional fully ionic model with uniform (non-ischemic) cell types, the earliest activating cells can repolarize earlier than the latest activating cells, resulting in a gradient of transmembrane potentials during the ST segment that in turn causes ST segment depression or elevation. Aslanidi et al. [5] also simulated ischemia, and found that ST segment deviations, measured with the PQ segment as a baseline, may also be caused by an abnormal sequence of repolarization and changes in ischemic cells' resting potential; hyperkalemia was the major contributor to these ischemia induced changes.

This work builds on the possibility of activation/repolarization sequence induced ST segment changes advanced by Aslanidi et al. [5] by showing that, in a three dimensional model, the widespread negative potentials throughout the inferior torso during the early ST segment can be simulated by modeling the earlier activating endocardium with lower transmembrane potentials than the later activating epicardium. The specific location of the potential minimum on the lateral left precordium (near 12 lead EKG lead V5) can result from a transmembrane potential gradient *across the epicardium*, with a site of later epicardial activation having lower extracellular potentials (ST depression) than the site of earliest epicardial activation.

This study also aims to offer a plausible explanation of how the early ST segment left precordial minimum *could *move to different location over the course of the ST segment, as reported by Kubota et al. [6]. As shown in the right hand panel in Figure 1, during the late ST segment, the potential minima in the case of LAD, RCA and LCX occlusions were over the anterior left precordium, anterior/inferior torso and the back respectively. Consistent with the solid angle theory, the location of these late ST segment minima were generally somewhere over the compromised perfusion bed. The modeling results presented here will show that the movement of the minima may be explained by a relative reduction in the transmembrane potential gradient across the epicardium during the ST segment, and an ischemia induced relative increase in the transmembrane potential gradient across the endocardium during that time.

## Methods

Computer simulations were performed with a 3-D monoventricular heart model. For each of the three types of single vessel disease (LAD, LCX and RCA), two single points in time, corresponding to the early and late ST segments, respectively, were simulated by assigning transmembrane potentials (TMPs) to each node within the model in such a manner as to match clinically observed torso potential distributions. A qualitative sensitivity analysis was carried out to show how different TMP distribution parameters (e.g. magnitude of the voltage drop across the epicardium) affected torso potential distributions.

### Model setup

As mentioned, a monoventricular model was adopted. Monoventricular models have elucidated important features of cardiac function[7,8]. A monoventricle was used because it has a single endocardial surface and a single epicardial surface, which greatly simplified the process of assigning TMP distributions throughout the heart. The Discussion section includes an analysis regarding how the lack of a septum might have affected the results.

To provide a realistic geometry, the ventricle was based on the epicardium of the Auckland canine heart[9] and the endocardial surface was a scaled version of the epicardium. The baseline scaling resulted in an approximate wall thickness of 1 cm. The mono-chamber was filled with blood, and the heart was immersed in a realistic torso according to a coupling method described elsewhere[10]. An electrically insulating layer was interposed between the ventricles and an atrial "cap" to simulate the atrioventricular junction. The boundary conditions between the myocardium and conductive fluid (intraventricular blood or torso) were that the extracellular potential was continuous, and no intracellular current could flow from the heart tissue into the conductive fluid.

The computer model solved the equation governing the passive flow of current in the heart, according to the bidomain theory, given a distribution of transmembrane potentials. In particular, the computer solved for the extracellular potential (*V*_*e*_) in the elliptical equation ∇ • (σ_*i *_+ σ_*e*_)∇*V*_*e *_= -σ∇*V*_*t *_where σ_*i *_and σ_*e *_are the intracellular and extracellular conductivity tensors, and V_*t *_is the TMP of each node in the myocardium. The detailed computational scheme was described previously[11]. Briefly, the heart was meshed by dividing it into 40 nested shells from endocardium to epicardium. The space in between any two consecutive shells was meshed with hexahedral elements parameterized by polar and azimuthal angle divisions in a spherical coordinate system. The Galerkin finite element method was used to solve the elliptical equation for extracellular potential, and Gauss quadrature was used to perform the numerical integration to create the finite element stiffness matrix. The resulting linear system of equations was solved using the pre-conditioned conjugate gradient method ('pcg.m' in Matlab ver 7.#, Mathworks, Inc., Natick, MA) with an incomplete (0 fill in) Cholesky decomposition as a preconditioner.

Fiber angles rotated linearly[8,12] from approximately -60 degrees to +60 degrees from epicardium to endocardium. Fibers were constrained to lie on the surface of each heart shell. Conductivity was assigned to blood and heart tissue as shown in Table 1. These values are generally in line with published estimates[13] although a rather larger value for the transverse intracellular conductivity was chosen in order to reduce anisotropy, which resulted in better matches with experimental data.

**Table 1:** Conductivity Values

Parameter	symbol	value
longitudinal extracellular conductivity	σ_el_	0.13S/m
longitudinal intracellular conductivity	σ_il_	0.13S/m
transverse extracellular conductivity	σ_et_	0.04S/m
transverse intracellular conductivity	σ_it_	0.02S/m
blood conductivity	σ_b_	0.66S/m
torso/atrial cap conductivity	σ_t_	0.33S/m

### Transmembrane potential distribution

As mentioned, the primary aim of the present study was to examine plausible mechanisms underlying clinical data pertaining to the ST segment associated with ischemia. To achieve this aim, TMP distributions were derived that produce body surface potential patterns that generally matched those seen in clinical data[4,6]. The relationship between intra-cardiac potentials and body surface potentials is non-unique [14]. Given this non-uniqueness, various assumptions were made to constrain TMP distributions in a realistic manner, based on the normal activation and recovery sequences of the heart and the changes to endocardial repolarization associated with subendocardial ischemia.

The first assumption that was made to simplify the problem was that TMPs varied linearly from the endocardium to the epicardium. To test the effect of this assumption, an alternative TMP distribution was simulated in which there were no transmural TMP gradients within the inner 2/3 of the heart wall ("inner layer") and no transmural TMP gradients within the outer 1/3 of the heart wall ("outer layer"), so that the entire transmural TMP gradient existed at the interface between the inner and outer layers. The resulting sharp TMP gradient acted to change the magnitude of epicardial and torso potential patterns, but, importantly, did not alter the patterns of the distributions.

The second assumption was that the TMPs along any surface (e.g. the epicardium, endocardium or any "surface"/shell in between) had only one local maximum and one local minimum. This is possibly an oversimplification, but is nonetheless reasonably consistent with endocardial and epicardial extracellular measurements by Li et al [1].

The location of the ischemic core region for each of the three diseased arteries (LAD, LCX and RCA) was selected by dividing the heart into approximately 70 sections and computing the torso potentials associated with a transmural TMP gradient in each section. Specifically, each section extended from the endocardium to the epicardium, and the transmural TMP gradient of a particular section was set such that the epicardium was at 1 and the endocardium was at 0. Lateral current sources between the selected section and all other sections were turned off, so that only the transmural TMP gradient across the selected section produced torso potentials. The core of the ischemic regions for the respective LAD, LCX and RCA simulations was selected as the section that produced the best matches for the location of the corresponding late ST segment minima shown in the right hand panel in Figure 1. (In the case of the LAD simulations, the desired minimum was on the left precordium, not on the upper right shoulder.)

For the early ST segment epicardial potentials, the location of the epicardial minimum (TMP maximum) was selected by again dividing the heart into approximately 70 sections and computing the torso potentials associated with a TMP gradient between a selected section and all other sections. Specifically, the TMP of a particular section was set to 0, and the TMP of all remaining sections was set to 1. The location of the epicardial minimum (TMP maximum) was selected as the section that produced a very focused minimum on the left precordium, near the area of precordial lead V5, in accord with clinical studies[3,15]. In reality, the latest to activate epicardial region is probably more postero-basal than the area located by the procedure mentioned above[16]. To test to the effect on torso potentials of a more postero-basal epicardial minimum (TMP maximum), a simulation was run with the epicardial minimum at a postero-basal location.

The same epicardial TMP pattern was used for late ST segment LAD, LCX and RCA simulations. This pattern was selected by setting the endocardial TMP distributions for LAD, LCX and RCA occlusions, respectively, and then searching for a single epicardial TMP distribution that produced good qualitative matches with the late ST segment torso potentials reported by Kubota et al. [6] for all three arteries. Again, the search was performed by dividing the heart into sections.

The epicardial maximum (TMP minimum) for both the early and late ST segment was set in the area of the anterior right ventricle in accordance with experimental data[17,18].

Given the location of an endocardial or epicardial TMP maximum or minimum, the TMP distribution for an entire surface (e.g. the epicardium) was selected by experimenting with various smoothed TMP distributions having potential extrema in the chosen locations.

**Figure 2:**
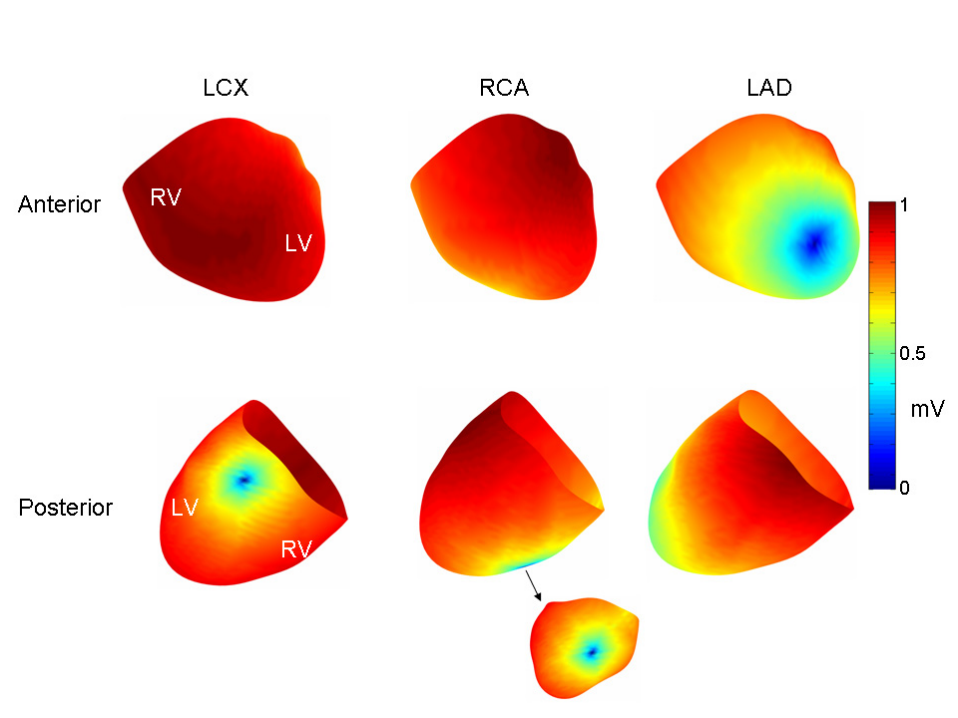
Endocardial transmembrane potential patterns. Endocardial TMP patterns for LCX, RCA and LAD ischemia, respectively. The TMP's are normalized so that they range between [0,1]. The core of the ischemic region is indicated by dark blue coloration.

**Figure 3:**
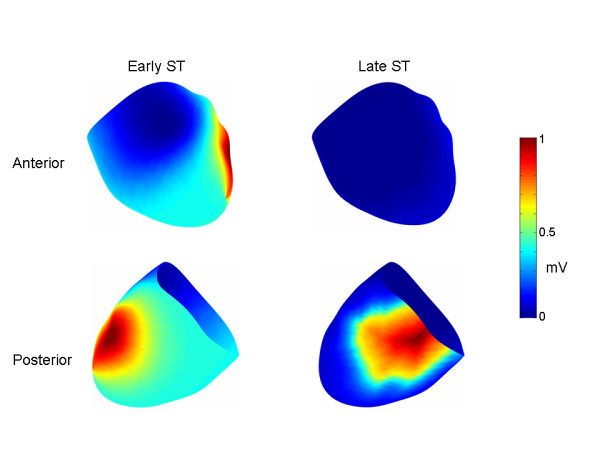
Epicardial transmembrane potential patterns. The same epicardial TMP patterns were used for all three ischemic regions (i.e., LCX, RCA and LAD) for early and late ischemia, respectively. The TMP's are normalized so that they range between [0,1]. The early ST segment epicardial TMP distribution is characterized by an anterior to lateral epicardial TMP gradient, while the late ST segment epicardial TMP distribution is characterized by an anterior to posterior TMP gradient.

The above mentioned procedure resulted in endocardial and epicardial TMP patterns shown in Figures 2 and 3. The TMPs for these patterns were normalized to values between [0,1] mV and then scaled to produce reasonable quantitative matches between generated torso potentials and clinical data. The Results section will show the torso potentials produced by different scaling factors for both the epicardium (S_epi_) and the endocardium (S_endo_).

In addition to the scaling of the epicardial and endocardial potentials, the overall transmural TMP difference between epicardium and endocardium influenced the results. For example, if the scaled endocardial TMPs ranged from 0 mV to 20 mV, and the scaled epicardial TMPs ranged from 0 mV to 5 mV, adding an offset of 5 mV to the scaled epicardial TMPs resulted in epicardial TMPs that ranged from 5 mV to 10 mV. Thus, three factors, epicardial scaling (S_epi_), endocardial scaling (S_endo_) and the overall offset between epicardial and endocardial TMPs, were found to alter torso potentials. Since only the difference between TMPs matters, not the absolute TMP values, 0 mV was chosen as a convenient baseline minimum TMP, which always occurred at the core of the subendocardial ischemic region. The three factors, S_epi_, S_endo _and the endocardial/epicardial offset, were iteratively adjusted to produce the best matches (determined by visual inspection) between simulated and measured torso potentials. Table 2 shows the final values for these factors.

**Table 2:** Scaling factors and epicardial/endocardial offset

	LAD		LCX		RCA	
	Early	Late	Early	Late	Early	Late

S_epi_	13	3	13	5	13	3
S_endo_	10	22	10	20	10	15
Offset (mV)	6	6	6	17	6	12

Key: Early = Early ST segment; Late = Late ST segment.

The values of these factors correspond generally to a decrease in the epicardial TMP gradient between the early and late ST segments, an increase in the endocardial TMP gradient between the early and late ST segments, and an overall increase in transmural TMP gradient between the early and late ST segments.

### Sensitivity analysis

A sensitivity analysis was performed to relate torso potentials to changes in epicardial scaling (S_epi_), endocardial scaling (S_endo_), and the overall epicardial/endocardial offset. The endocardial LAD, LCX and RCA TMP distributions and the early ST epicardial TMP distribution, which were obtained in the manner described in Section B above, were chosen as the distributions for performing the sensitivity analysis. For each of the LAD, LCX and RCA TMP distributions, three sets of sensitivity simulations were performed. In each of the three sets, one of three factors (S_epi_, S_endo _and epicardial/endocardial offset) was varied while the other two were held constant.

In addition, in order to distinguish between epicardial TMP gradient effects and transmural TMP gradient effects, simulations were run with a TMP distribution characterized by endocardial TMPs that were uniformly lower (more repolarized) than epicardial TMPs. There were no TMP gradients across the endocardium or across the epicardium.

Another simulation was run in which there was no transmural TMP gradient. The TMP gradient across all surfaces (e.g. endocardium, epicardium and all layers therebetween) was identical to the epicardial TMP distribution shown in Figure 3 for the early ST segment.

## Results

**Figure 4:**
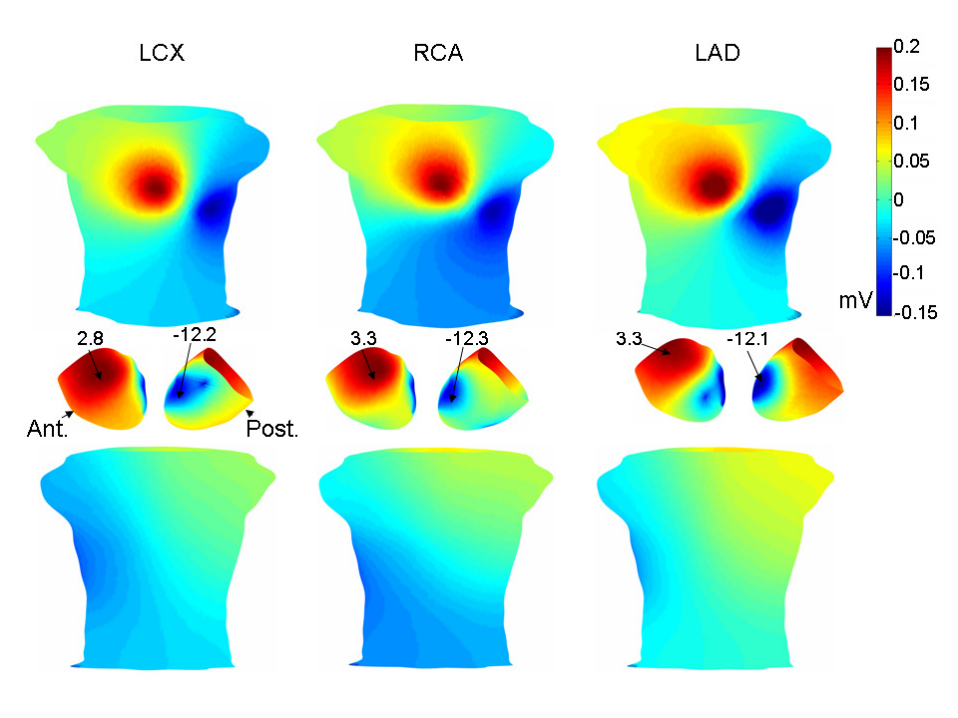
Early ST segment torso potential distributions. Torso potential distributions (anterior top row, posterior bottom row) corresponding to the early ST segment in the case of LCX, RCA and LAD occlusions, respectively. The colorbar on the right indicates the voltages for all of the torso distributions. In the middle row are anterior (left) and posterior (right) views of the TMP difference between the endocardium and the epicardium. See text for additional details.

The top and bottom panels in Figure 4 show early ST segment torso potentials corresponding to the LAD, LCX and RCA simulations, respectively. As shown in the Figure, the torso potential distributions are generally similar, with a potential maximum in the mid-anterior sternum and a potential minimum in the lateral left precordium. The upper right torso is characterized by positive potentials.

The middle panel in Figure 4 shows each of the endocardial TMPs minus the corresponding epicardial TMP, projected onto the epicardium. The maximum and minimum voltages (mV) are indicated. (The torso color bar does not apply to the middle panel potential distributions.) In each case, the greatest transmural TMP gradient is in the area of the lateral left free wall.

**Figure 5:**
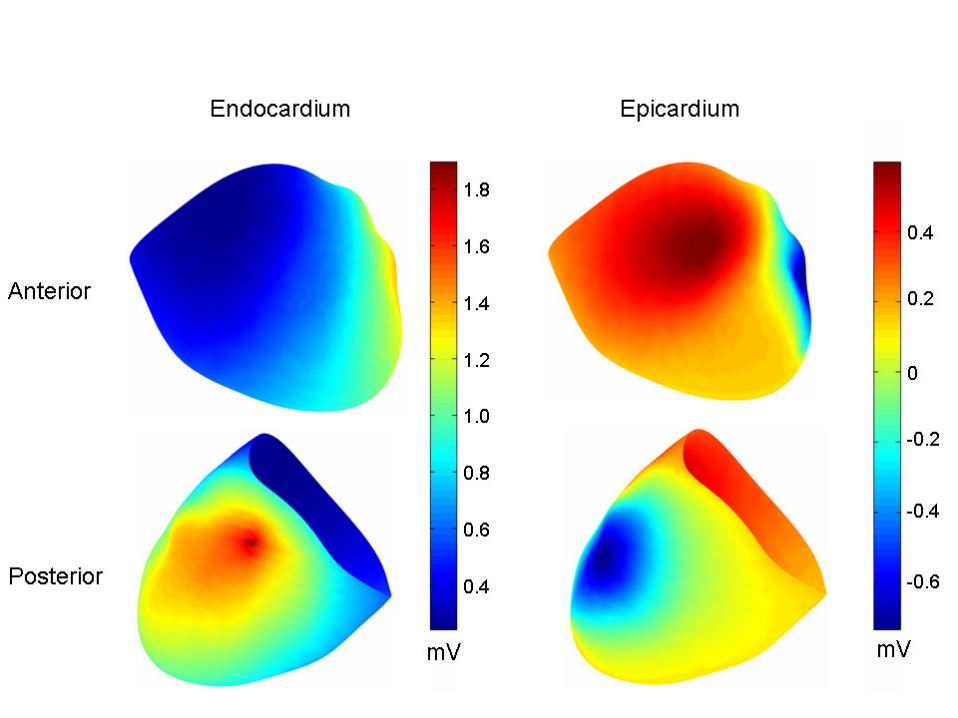
Early ST endocardial and epicardial extracellular potential distributions for LCX. On the endocardium, the maximum is located within the core of the ischemic region. On the epicardium, the minimum is located over the lateral left free wall, not over the core of the ischemic region.

Figure 5 shows the epicardial and endocardial extracellular potential distributions for the LCX occlusion. On the endocardium, the potential maximum is centered on the ischemic area, as expected. On the epicardium, the potential minimum is located on the lateral left wall; the minimum is not centered over the ischemic region. The epicardial potential distributions in the case of LAD and RCA ischemia were very similar to LCX ischemia. The endocardial potential maxima in the case of the LAD and RCA occlusions were also centered on the ischemic region.

**Figure 6:**
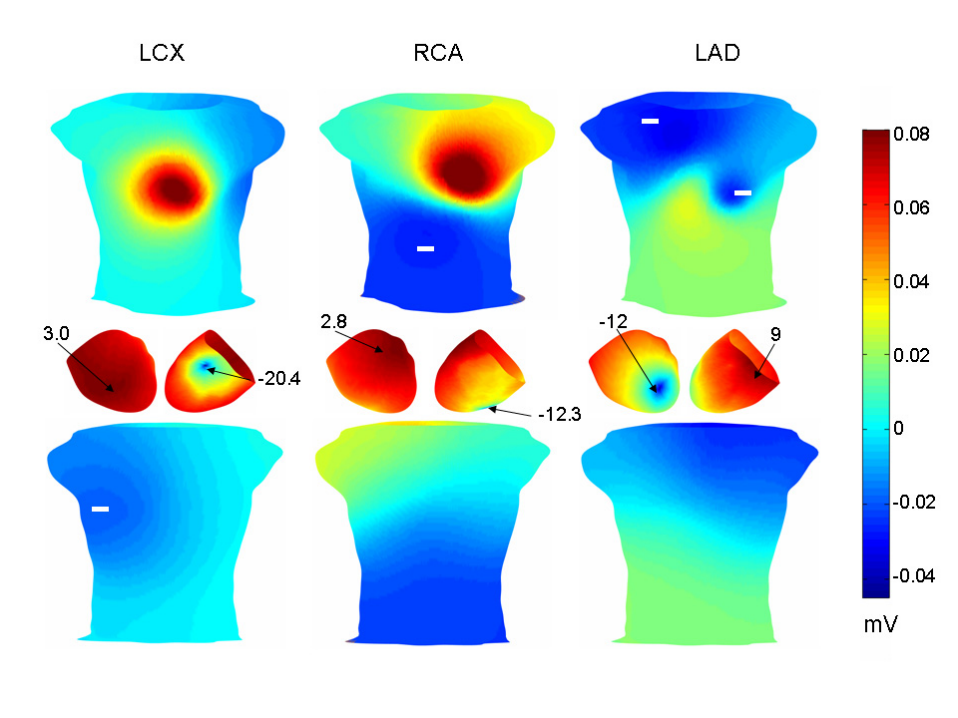
Late ST segment torso potentials. Torso potential distributions (anterior top row, posterior bottom row) in the case of LCX, RCA and LAD occlusions, respectively. The colorbar on the right indicates the voltages for all of the torso distributions. As in Figure 5, in the middle row are anterior (left) and posterior (right) views of the TMP difference between the endocardium and the epicardium.

Figure 6 shows the torso potentials for the late ST segment corresponding to the LAD, LCX and RCA simulations, respectively. In the case of the LCX simulation, the minimum was located on the left back, the maximum was in the same location as in the early ST simulations, and small positive potentials characterized the majority of the remaining anterior torso and portions of the posterior torso. For the RCA, the minimum was on the lower right anterior torso, and there was a general superior to inferior pattern of positive to negative potentials. For the LAD, there were two minima, one over the anterior left ventricle and another over the right shoulder. Positive potentials otherwise characterized most of the rest of the inferior torso.

**Figure 7:**
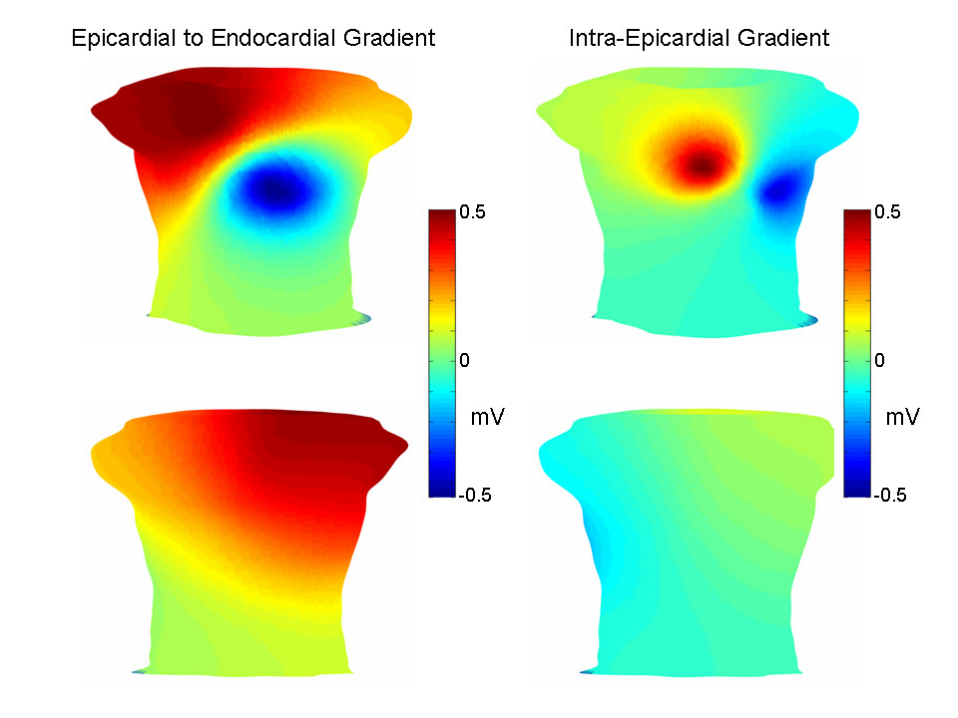
Sensitivity analysis. Torso potential distributions (anterior top row, posterior bottom row) corresponding to a constant trasnmural TMP gradient (left) and no TMP gradient (right). Torso potentials were normalized to range between -0.5 mV and 0.5 mV. See text for additional details.

The sensitivity analysis showed that both a global transmural TMP gradient and a global TMP gradient across the epicardium (intra-epicardial TMP gradient) were necessary to generate early ST segment torso potentials consistent with the results of Hanninen et al. [4] (Figure 1, left panel). Specifically, the epicardial/endocardial TMP offset controlled the magnitude of the lower left to upper right body surface potential distribution aligned with the arrow in the left panel in Figure 1. This pattern is similar to that shown in the left hand panel in Figure 7, which was obtained by applying only a transmural TMP gradient. More specifically, in the constant TMP gradient case in the left hand panel in Figure 7, the endocardial TMPs were lower than the epicardial TMPs by a fixed amount.

Given a particular epicardial/endocardial TMP offset, the epicardial scaling (S_epi_) needed to be set within a fairly narrow range (12–14) to create a minimum over the lateral left side of the torso, as desired (Figure 1, left panel). Given the epicardial/endocardial offset and S_epi, _the torso potentials were not significantly affected by the endocardial TMP distribution, as indicated by the similarity of LAD, LCX and RCA occlusions (Figure 4), provided that the endocardial scaling (S_endo_) was somewhat smaller than the epicardial scaling (less than 11).

Without any epicardial/endocardial TMP offset, a minimum in the lateral left precordium was obtained by applying the epicardial TMP distribution to all heart layers (i.e. no transmural TMP gradient), as shown in the right hand panel in Figure 7. In particular, the TMPs on each heart layer/shell followed the epicardial pattern shown in the left hand panels in Figure 3. Although the pattern shown in the right hand panel in Figure 7 matches the location of the clinically reported lead V5 area minimum and does result in diffuse inferior negative potentials, it does not produce a lower left to upper right shoulder torso potential distribution aligned with the arrow in the left hand panel in Figure 1.

In sum, both an appropriate endocardial/epicardial offset and an intra-epicardial TMP gradient were required to match the main features of the early ST segment clinical and experimental data. To obtain the late ST segment potentials reported by Kubota et al. [6], all of the factors (endocardial/epicardial offset, S_epi _and S_endo_) had to be fairly close (within 1 or 2 units) of the final values.

**Figure 8:**
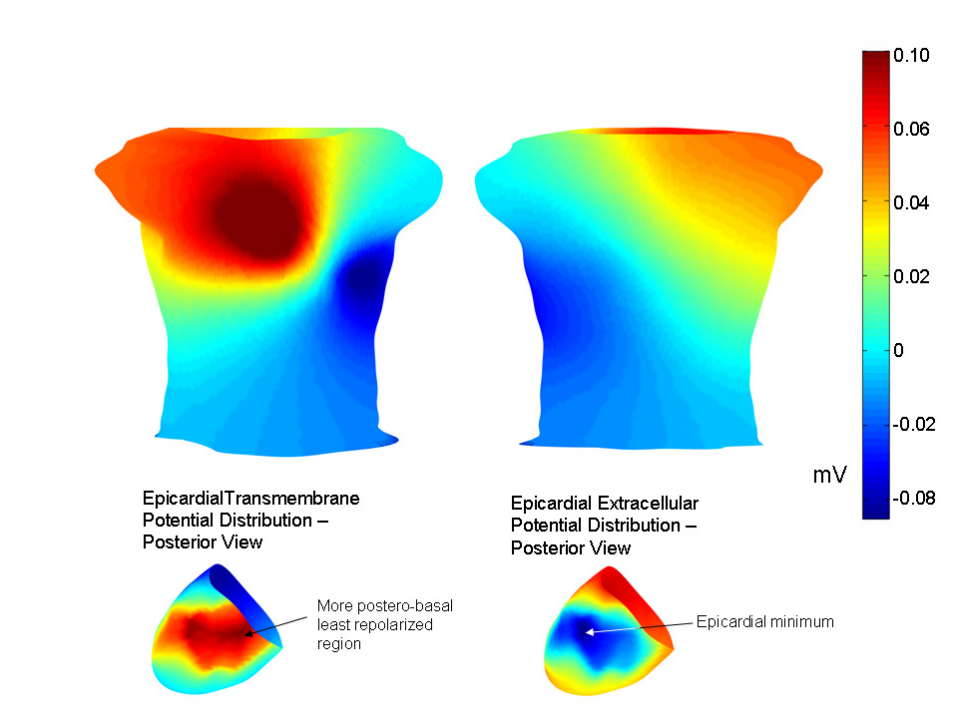
Modified epicardial TMP distribution. A more postero-basal epicardial region was chosen to correspond to the site of the least repolarized epicardial region (epicardial minimum), as shown in the lower panel. The endocardial TMP distribution was the early ST segment LCX endocardial TMP distribution. The anterior and posterior torso potential distributions are shown above. The pattern is broadly similar to that shown in Figure 4.

Figure 8 shows early ST segment torso potentials associated with a more postero-basal epicardial minimum (lower panel) than the minimum shown in Figure 3 (left panels). The LCX endocardial TMP distribution was used for this simulation. The torso minimum is still near lead V5 and the torso potentials are broadly similar to those in Figure 4. In the simulations corresponding to Figure 8, S_epi _= 13, S_endo _= 10 and the epicardial/endocardial offset was set at 6.

## Discussion

### Overview

One of the major findings of this work is that diffuse left/inferior early ST segment depression, with a minimum near precordial lead V5, can be simulated (Figure 4) by applying a global intra-epicardial TMP gradient (Figure 3) and a global transmural TMP gradient, with the endocardium generally more repolarized than the epicardium (Figure 4, middle panel). The endocardial extracellular potential maximum occurred in the ischemic core region (Figure 5) but the endocardial potential pattern left no obvious imprint on the body surface potentials since the above mentioned global TMP gradients dominated the potential distribution on the torso. Another of the central findings of this work is that a reduction in the epicardial TMP gradient and an increase in the endocardial TMP gradient can result in late ST segment body surface potential minima over the ischemic core regions in the case of single artery LAD, LCX and RCA disease (Figure 6).

The above mentioned early and late ST segment TMP distributions could have a biophysical basis. During the early ST segment, sub-endocardial ischemia and/or the activation/repolarization sequence, including delayed activation of the epicardium due to slower transmural conduction through ischemic subendocardium[19], may result in the endocardium being generally more repolarized than the epicardium. Thus, the endocardium is characterized by relatively positive extracellular potentials compared to the epicardium, which tends to cause positive potentials over the right shoulder and negative potentials towards the left and inferiorly (Figure 4). On the epicardium, relatively late activating tissue in the lateral left free wall may have relatively high (depolarized) TMPs since this tissue has had less time to repolarize then the earlier activated cells. These higher TMPs in the lateral left epicardium result in an epicardial TMP gradient because the earlier activated epicardial cells have lower TMPs. Higher TMPs in the lateral left ventricle also cause a relatively strong transmural TMP gradient there because the underlying endocardium has repolarized relatively more due to electrotonic coupling with ischemic subendocardium and/or activation/repolarization sequence effects. These two TMP gradients, intra-epicardial and transmural, combine to induce a very large negative potential within the left ventricular free wall and the nearby torso (precordial leads V5/V6).

To the extent early ST intra-epicardial and transmural TMP gradients are affected by heart rate, it may be expected that higher heart rates could cause early ST segment depression in healthy subjects. The accelerated repolarization associated with higher heart rates would cause the earliest to activate epicardial cells (anterior right ventricle) to partially repolarize while the propagation wave is proceeding toward the lateral free wall. For similar reasons, the earlier activating endocardium would generally tend to be more repolarized by the time the epicardial lateral free wall depolarizes. Consequently, even in a non-ischemic heart, the lateral free wall may be negative during the early ST segment, which is consistent with exercise induced J point depression in leads V5/V6. Thus, activation/repolarization sequence effects could explain aspects of ST depression, as pointed out by Aslanidi et al. [5]. With regard to the early ST segment at higher heart rates associated with ischemic subjects, relatively greater ST depression at a given heart rate may occur due to ischemia induced propagation delays and/or a global acceleration of subendocardial repolarization.

According to the above argument, the lateral left free wall is characterized by relatively negative extracellular potentials during the early ST segment due to relatively late activation of this area. It follows that the latest to activate epicardial regions, which are likely in the postero-basal region[16], would be associated with the most negative extracellular potentials during the early ST segment. However, this does not mean that the minimum on the body surface is on the back, directly over the postero-basal region, the most negative epicardial region. Because the posterior region of the heart is further away from the torso surface than the left lateral free wall, the negative extracellular potentials on the lateral left free wall leave a larger imprint on the body surface than the negative posterior potentials, as shown in Figure 8. Thus, even though the epicardial extracellular minimum may be in the postero-basal region, the torso minimum may not be over this area but rather may be over the lateral left free wall.

Returning to the general theory regarding the effect of the activation/repolarization sequence on torso potentials, it is possible that over the course of the ST segment, the latest to activate epicardial regions "catch up" somewhat to the earlier to activate epicardial regions, thereby decreasing the epicardial TMP gradient. The reduction in the epicardial TMP gradient can unmask the location of the ischemic region for two reasons. First, this reduction means that the strongest transmural TMP gradients overlie the ischemic region (i.e. the strongest transmural TMP gradients are no longer in the area of the later activating epicardial regions), as may be seen by comparing the transmural TMP differences in the middle row of Figure 4 with the transmural TMP differences in the middle row of Figure 6. Second, the reduction of the intra-epicardial TMP gradient reduces intra-epicardial current/voltage sources, which tend to make the later activating epicardial regions more negative during the early ST segment. In the simulations, the late ST epicardial extracellular potential distribution pattern did not mirror the epicardial TMP distribution pattern, shown in Figure 3, right panel, but instead exhibited a potential minimum over the ischemic region. In contrast, the early ST epicardial potential distribution largely mirrored the epicardial TMP distribution pattern (compare Figure 3, left panel with Figure 5, right panel).

While the epicardial TMP gradient may be decreasing during the ST segment, the TMP dispersion across the endocardium may be increasing, as the ischemic cells repolarize more rapidly than the non-ischemic endocardial cells. The removal of the masking epicardial TMP gradients along with the relative decrease in the ischemic cells' TMPs result in the ischemic cells having the relatively lowest TMPs compared to the overlying epicardium. This in turn results in a potential minimum on the torso in an area over the ischemic region, as would be predicted by solid angle theory[1].

Finally, although the epicardial TMP gradients are much smaller in the late ST segment compared to the early ST segment, an anterior to posterior epicardial TMP gradient helped to generate late ST torso potentials that matched the data of Kubota et al. [6] for the LCX occlusion. An anterior to posterior epicardial TMP gradient during the late ST segment is consistent with an anterior to posterior T-wave epicardial TMP gradient that has been predicted with an inverse solution model[20]. An anterior to posterior epicardial TMP gradient during the late ST segment is also consistent with data that shows the postero-basal region is the latest epicardial region to activate[16]. However, it is possible that the (small) anterior to posterior epicardial TMP gradient required in the current study reflects limitations of the model rather than a real biophysical phenomenon.

### Comparison with experiment/prior work

The central aim of this study was to derive TMP distributions that matched the patterns clinically observed by Hanninen et al. [4] (early ST segment) and Kubota et al. [6] (early and late ST segment). In the case of the early ST segment, the locations of the maxima and minima in the simulations approximated to the maxima and minima locations reported by Hanninen et al. Since the conductivity values and/or TMP voltages could simply be scaled, matching the torso potential magnitudes was not considered to be particularly important. Nonetheless, for purposes of comparison, the total voltage drop across the torso was between 0.25 mV–0.35 mV in the simulations, which conforms well to the 0.2 mV–0.25 mV implied by the figures in Hanninen et al. [4]

In the case of the late ST segment, the locations of the minima were very close to those reported by Kubota et al[6]. Although Kubota et al. reported some inter-subject differences, the minima (see Figure 1) of all subjects moved in the same general directions in the case of LAD, RCA and LCX occlusions, respectively. The model presented here successfully matched this movement of potential minima. Also, in the case of the LAD occlusion, the model matched the feature of a second minimum over the right shoulder. In the simulations, the magnitudes of the late ST potentials, more particularly the total voltage drop across the torso, was approximately half the total voltage drop during the early ST segment. The voltages reported by Kubota et al. were either in this range or somewhat larger by about 0.05–0.1 mV.

Li et al. [1] separately constricted the LAD and LCX in an open chest sheep preparation, and recorded (early) ST segment extracellular potentials from both the epicardium and the left ventricular endocardium. The potential maximum on the endocardium was located in the ischemic region in the case of both LAD and LCX occlusions, whereas the epicardial minimum was in the same area of the lateral left wall in the case of both LXC and LAD occlusions. The entire endocardium was generally at a higher potential than the entire epicardium, at least according to some of the data reported by Li et al. Finally, again according to at least some of the reported Li et al data, the overall epicardial voltage drop was greater than the overall endocardial voltage drop, which suggests that epicardial voltage/current sources contributed significantly to the epicardial potentials.

Consistent with the Li et al. work, in the present study, the endocardial maximum was located in the ischemic region and the epicardial minimum was in the area of the lateral left free wall (see Figure 5 for the case of LCX occlusion). Further, as in the Li et al. study, a large portion of the endocardium outside of the ischemic region was at a higher extracellular potential than the epicardium. In sum, the present study matches many of the important features of the Li et al. study.

In the context of a human clinical study, Fox et al. [21] reported that torso potentials after exercise did tend to localize the ischemic region. In a related study, Fox et al. [22] found that the area of ST segment depression grew steadily after the cessation of exercise, then eventually diminished. It is possible that non-ischemic tissue recovers from exercise more rapidly than the ischemic tissue, and that healthy endocardial tissue recovers from exercise more rapidly than epicardial tissue. If so, then the post-exercise early ST segment would appear more like the late ST segment at higher heart rates. However, in the studies of Fox et al., single vessel LAD disease caused ST depression in a more superior region of the torso compared to the location of torso minima reported by Kubota et al. [6] Thus, although the results of Fox et al. are not necessarily in conflict with the theories advanced in the present work, these results require further analysis in light of these theories.

A computer study of ischemia by Arola et al. [23] matched the general pattern of negative potentials on the lower left precordium and positive potentials on the upper right precordium by setting an ischemic region as almost half the size of the heart, including the left ventricular inferior apex and the right chamber. Because the study of Arola et al. [23] involved assigning dipoles to an ischemic area, the relationship between transmembrane potentials and extracellular potentials was not explored.

### Limitations

The present model extends the work of prior full heart models [1-3] by relying upon a model that is unique in three main respects. First, the model incorporates global epicardial TMP gradients into a model of subendocardial ischemia. Second, the model incorporates a global endocardial to epicardial TMP gradient into a model of subendocardial ischemia. Third, the model shows how physiologically realistic changes in these gradients can result in late ST segment potentials reported by Kubota et al. [6] While the assignment of endocardial and epicardial TMPs produced the desired torso potential distributions, which has not been possible with prior models, there are several limitations which should be noted.

First, given the non-unique nature of the relationship between intra-cardiac potentials and body surface potentials[14] which generally characterizes inverse solutions, no definitive proof can be offered with respect to the actual genesis of the clinically observed body surface potentials in conditions of ischemia. For example, relatively complicated epicardial potential patterns with multiple maxima and minima may correspond to a relatively simple torso pattern with just one maximum and one minimum[18]. As a additional example, in the present simulations, the torso minimum near lead V5 was produced with two different types of epicardial TMP distributions (compare the epicardial TMP distribution in Figure 3, left panel, with the distribution in Figure 8). No attempt was made to find other TMP distributions that would result in a minimum at this location. Thus, the exact nature of the epicardial and transmural TMP gradients that produce the torso minimum near lead V5 remains an open question. Similarly, the late ST segment epicardial TMP gradient employed in the present work may be substantially different than the actual epicardial TMP gradients that underlie the late ST segment patterns observed by Kubota et al. [6]

On the other hand, other aspects of the present study would seem to have a reasonably firm grounding, notwithstanding the issue of non-uniqueness. In particular, the torso difference maps (see Figure 1, left panel) reported by Hanninen et al. [4] that manifest a large scale lower left torso to upper right shoulder negative to positive pattern would seem to be most easily explained by an overall TMP difference between endocardium and epicardium. Further, the relatively simple dipolar late ST segment patterns observed by Kubota et al. [6], especially in the case of single vessel LAD and RCA disease, were most likely caused by a core endocardial ischemic region with lower TMP's than the rest of the endocardium; this explanation is consistent with the solid angle theory of ischemia[1]. (In the case of the LCX, the posterior minimum reported by Kubota et al. could be generated by the plausible combination of a strong epicardial TMP gradient with a pattern like that shown in Figure 3, right panel, coupled with a relatively small endocardial TMP gradient.) The present work showed how the early ST torso potential pattern can change to the late ST torso potential pattern in a manner consistent with the process of the repolarization dynamics of both healthy and ischemic tissue. The non-uniqueness problem does not vitiate this demonstration.

The lack of a septum in the 3-D model may have affected the simulation results. There are two types of TMP gradients that may involve the septum: a surface like gradient akin to the intra-epicardial and intra-endocardial TMP gradients used in the simulations, and a transmural like gradient across the septum from the left ventricular side to the right ventricular side. Regarding a surface type gradient, the presence of highly conductive ventricular blood adjacent to the septum suggests that transmembrane gradients deep within the septum have very small imprints on the epicardium[1].

A transmural like gradient across a large portion of the septum, however, could conceivably affect torso potentials. Such a septal gradient has been posited as the cause of the normal q wave, i.e. negative potentials in lead V5. If the repolarization sequence follows the activation sequence, then the septal TMP gradient during repolarization would tend to cause positive potentials in lead V5 since the repolarization polarity is opposite to the depolarization polarity (i.e. earliest activating cells are negative whereas earliest repolarizing cells are positive). Positive potentials in lead V5 are the opposite of the negative potentials observed in that lead in connection with subendocardial ischemia and/or high heart rates. Thus, a model in which ischemia induces early repolarization of the left ventricular area of the septum, which would be consistent with LAD and LCX disease, would not help to explain clinical body surface data pertaining to subendocardial ischemia.

In sum, although the lack of a septum may have affected torso potential distributions, the major aspects of at least some clinically observed torso patterns[4,6] are most easily explained by transmembrane potentials throughout the heart outside of the septum. It is possible that a septum may help to explain other clinically observed patterns.

The use of a homogenous torso almost certainly influenced the body surface potentials produced by the model. A more realistic torso model, with lungs, skeletal muscle and fat, may correspond to different TMP distributions than those that were required to generate torso potentials that matched the clinical body surface data. However, the overall pattern of results would very likely remain consistent with, and not alter the conclusions generated from, the model used in the present study.

The anisotropic nature of heart tissue somewhat complicates the simple picture of a region of negative potentials centered over a region of subendocardial ischemia. Briefly, anisotropy will tend to cause the epicardial area over the ischemic region to be positive (or less negative) than the area over the ischemic/healthy boundary[1,2,24]. (More strictly, unequal transverse and lateral conductivity ratios will cause this effect.) The actual potential distribution depends on the balance between the uniform source, which tends to cause a centered negative region over the ischemic region, and anisotropy. The effects of anisotropy in practice depend on, among other things, the actual conductivity of heart tissue and the TMP distribution[2,24,25]. According to the present simulations, which are consistent with the data of Kubota et al., these factors interact to cause a region of negative potentials over the ischemic region.

More generally, there are a host of uncertainties regarding model parameter values, ischemic TMP distributions and the like. To the extent there is uncertainty regarding parameter values, existing literature was relied upon to constrain these values in a realistic manner. Finally, a fully ionic model simulation would help to confirm many of the biophysical arguments advanced in this work.

## Conclusion

The present work has illustrated plausible mechanisms responsible for generating the body surface patterns that are often observed during ischemia. These mechanisms involve the roles of the activation sequence, the normal epicardial repolarization sequence, and the accelerating and resequencing of the endocardial repolarization sequence due to ischemia. All of these factors could give rise to certain TMP distributions that contribute to the observed body surface potentials when these are modeled using principles related to the solid angle theory. Solid angle theory may not strictly apply due to the effects of anisotropy, but nonetheless qualitatively explains much of the observed body surface potential patterns arising from subendocardial ischemia, at least according to the present model.

The global nature of some of the electrical changes implied by the current results is not entirely novel[26] but is nonetheless controversial. One possibility for ischemia induced global changes is that even single vessel coronary artery disease is associated with an increase in left ventricular end diastolic pressure[26]. Greater end diastolic pressure may tend to globally depolarize myocytes via stretch activated ion channels, which in turn would tend to globally decrease action potential duration (i.e. increase repolarization speed). Also, electrical coupling between cells may act to spread the electrical effects of ischemia over a wide region. More generally, it is possible that the heart is adapted to repolarize synchronously in layers (i.e. outer layer/epicardium first and then the inner layer/endocardium), so that within a layer, there is a tendency of the cells to react as a group. Mechanical feedback could conceivably play a role in this system.

## Abbreviations

TMP = transmembrane potential distribution
